# Der Kongress Armut und Gesundheit 2022 vor dem Hintergrund der aktuellen Krisen

**DOI:** 10.1007/s00103-022-03577-0

**Published:** 2022-08-23

**Authors:** Maren Janella, Marion Amler, Julian Bollmann, Nicole Böhme, Claudia Czernik, Marina Martin, Julia Waldhauer

**Affiliations:** 1 Gesundheit Berlin-Brandenburg e. V., Berlin, Deutschland; 2 Abteilung für Epidemiologie und Gesundheitsmonitoring, Robert Koch-Institut, Nordufer 20, 13353 Berlin, Deutschland

**Keywords:** Public Health, Gesundheitliche Ungleichheit, COVID-19, Öffentlicher Gesundheitsdienst, Gesundheitsförderung, Public health, Health inequalities, COVID-19, Public health services, Health promotion

## Abstract

Der Kongress Armut und Gesundheit wird seit 1995 jährlich von Gesundheit Berlin-Brandenburg e. V. in Zusammenarbeit mit verschiedenen Kooperationspartner*innen ausgerichtet. Inzwischen gehört er zu den größten regelmäßig stattfindenden Public-Health-Veranstaltungen in Deutschland. Der Kongress versteht sich als eine Plattform für den Austausch zwischen Wissenschaft, Praxis, Zivilgesellschaft und Politik. Unter dem Motto „Was jetzt zählt“ wurden in diesem Jahr Aspekte gesundheitlicher Ungleichheit und Public-Health-relevante Fragestellungen vor allem vor dem Hintergrund der COVID-19-Pandemie und des Krieges in der Ukraine diskutiert. In über 100 digitalen Veranstaltungen mit mehr als 2000 Teilnehmenden, darunter ca. 500 Referierenden, fand ein vielfältiger Austausch statt.

## Abstract (en)

Since 1995, the Poverty and Health Congress has been organized annually by Gesundheit Berlin-Brandenburg e. V. in cooperation with various partners. It is one of the largest regular public health events in Germany. The congress is a platform for exchange between science, practice, civil society, and politics. In 2022, the motto was “What counts now.” Public health and health inequalities were discussed in the light of the SARS-CoV‑2 pandemic and the war in Ukraine. Professional exchange on various topics took place in over 100 digital events with more than 2000 participants, among them around 500 speakers.

Der Kongress Armut und Gesundheit (hier auch: der Kongress) wird seit 1995 jährlich von Gesundheit Berlin-Brandenburg e. V. in Zusammenarbeit mit verschiedenen Kooperationspartner*innen ausgerichtet (siehe auch Infobox 1).

Er gehört zu den größten regelmäßig stattfindenden Public-Health-Veranstaltungen in Deutschland. Der Kongress versteht sich als eine Plattform für den Austausch zwischen Wissenschaft, Praxis, Zivilgesellschaft und Politik. Unter dem Motto „Was jetzt zählt“ wurden vom 22. bis 24.03.2022 Aspekte gesundheitlicher Ungleichheit und Public-Health-relevante Fragestellungen in über 100 digitalen Veranstaltungen mit mehr als 2000 Teilnehmenden, darunter ca. 500 Referierenden, diskutiert.

Die programmleitende Struktur bildete auch in diesem Jahr die Ottawa Charta für Gesundheitsförderung der Weltgesundheitsorganisation mit ihren 5 Handlungsfeldern^1^ [2]. Unter den Eindrücken mehrerer Krisen – der noch immer andauernden COVID-19-Pandemie, des Ende Februar 2022 begonnenen Krieges in der Ukraine und der hierdurch in den Hintergrund gedrängten Klimakrise – formulierte Frau Dr. Gabriele Schlimper (Vorsitzende des Paritätischen Landesverbandes Berlin) in der Eröffnungsveranstaltung: „Die Themen Armut und Gesundheit gemeinsam zu diskutieren, ist gerade jetzt von besonderer Bedeutung.“

Der Bundesgesundheitsminister Prof. Dr. Karl Lauterbach und die regierende Bürgermeisterin von Berlin, Franziska Giffey, die jeweils die Schirmherrschaft für die Veranstaltung übernommen hatten, unterstrichen dies in ihren Videobotschaften. Franziska Giffey erklärte: „Es geht darum, welche sozialen Fragen in der Pandemie aufgeworfen wurden.“ Karl Lauterbach lenkte den Fokus auch auf den Öffentlichen Gesundheitsdienst (ÖGD): „Die Pandemie hat gezeigt, dass gesundheitliche Chancen und Risiken nicht gleich verteilt sind.“ Um Maßnahmen der Gesundheitsförderung so auszurichten, dass sie die Zielgruppen besser erreichen, solle die Stärkung des öffentlichen Gesundheitswesens in den Ländern und Kommunen weiter vorangetrieben werden. Diesem komme nicht nur im Infektionsschutz, sondern auch bei der Bekämpfung gesundheitlicher Ungleichheit eine bedeutende Rolle zu.

Prof. Dr. Rolf Rosenbrock (Vorstandsvorsitzender des Paritätischen Gesamtverbands und von Gesundheit Berlin-Brandenburg e. V.) formulierte in seinem Grußwort: „Die ökologische Wende wird die sozial bedingte Ungleichheit von Gesundheits- und Lebenschancen vergrößern, wenn es nicht gelingt, sie in eine wirklich sozial-ökologische Wende zu transformieren. Das passiert nicht von allein.“ Es gelte nun, „Koalitionen zu schmieden, die der sowohl im Katastrophen- als auch im Normalbetrieb wirksamen, immer weiteren Spreizung von Gesundheits- und Lebenschancen etwas Wirksames entgegensetzen.“ Er schloss seine einleitenden Worte mit einem Zitat von Jean-Paul Sartre: „Vielleicht gibt es schönere Zeiten, aber diese ist unsere.“ Es gelte, diese nun verantwortungsbewusst mitzugestalten. Die diesjährige Keynote hielt Frau Prof. Dr. Jutta Allmendinger, Präsidentin des Wissenschaftszentrums Berlin für Sozialforschung. Unter dem Eindruck der aktuellen Dauerkrisen benannte sie 4 Kernherausforderungen unserer Zeit: den Krieg in der Ukraine, die COVID-19-Pandemie, den Klimawandel und die Digitalisierung.

## Krieg in der Ukraine: Engagement für Frieden und Gesundheit

Die European Public Health Association (EUPHA) umriss Ende Februar 2022 die Auswirkungen des Krieges auf die Gesundheit in ihrem Policy Statement „Health is at stake in the Ukraine invasion“ [1]. Zusätzlich zu den unnötigen Todesfällen wird die Gesundheit der Menschen z. B. durch die Zerstörung der Infrastruktur, Vertreibung, wirtschaftliche Einbußen, prekäre Lebensbedingungen und die Einschränkung demokratischer Prozesse bedroht. Stefan Pospiech, Geschäftsführer von Gesundheit Berlin-Brandenburg e. V., stellte sich als Veranstalter hinter die Verurteilung der Gewalt und die Forderung, die Gewalttaten sofort zu beenden.

In einer Veranstaltung mit dem Titel: „Klima, Krieg und Gesundheit – Wie Mediziner*innen sich für Klimaschutz und Frieden einsetzen können“, stellte Frau Dr. Angelika Claussen (IPPNW) vor, wie sich Menschen aus Gesundheitsberufen beispielsweise im Rahmen der Organisation „Internationale Ärzt*innen für die Verhütung des Atomkrieges (IPPNW)“ oder „Health for Future“ zivilgesellschaftlich für Frieden und eine klimagerechte Gesellschaft einsetzen. Dabei ging sie auf die Wechselwirkungen der Themen ein und führte aus, dass gerade Menschen in Gesundheitsberufen aufgrund ihrer Kompetenzen und auch ihrer ethischen Grundsätze prädestiniert seien, hier Verantwortung zu übernehmen.

## COVID-19 und gesundheitliche Ungleichheit

### Forschung zur gesundheitlichen Ungleichheit in der Pandemie

Die COVID-19-Erkrankung sowie die Folgen der Eindämmungsmaßnahmen verlaufen in Deutschland und international entlang eines sozialen Gradienten. Erkenntnisse aus dem Verbundforschungsprojekt InHeCov „Socioeconomic inequalities in health during the COVID-19 pandemic“ (siehe auch www.inhecov.de) wurden im Panel „Soziale Determinanten: COVID-19 und gesundheitliche Chancengerechtigkeit in Deutschland“ vorgestellt. Forschungsbedarf wird hier u. a. bei Post-COVID in Bezug auf soziale Ungleichheiten gesehen. Während die ersten Infektionen in Deutschland hauptsächlich Menschen mit höherem sozialen Status betrafen, z. B. aufgrund von erhöhter Mobilität (Dienst- und Fernreisen), breitete sich das Infektions- und Sterbegeschehen im Zeitverlauf zunehmend in sozioökonomisch benachteiligten Gruppen aus, so dass diese schließlich am stärksten betroffen waren, wie Dr. Jens Hoebel (Robert Koch-Institut – RKI) in seinem Vortrag resümierte [3]. Jens Hoebel forderte, dass für den Infektionsschutz und die Planung präventiver Angebote die Lebens- und Arbeitsbedingungen stärker in den Blick genommen werden sollten. Ebenso müssten die sozialen Determinanten bei der Pandemieplanung berücksichtigt werden. Dies könne beispielsweise durch gezielte niedrigschwellige Test- und Impfangebote mit aufsuchendem Charakter geschehen.

Dr. Timo-Kolja Pförtner (Universität zu Köln) stellte die Auswertung von Individuendaten des GESIS Panel Special Survey on the Coronavirus Outbreak aus dem Jahr 2020 vor. Unter der Fragenstellung „Alles eine Frage der Bildung?“ wurden aus den Daten Erkenntnisse zum Präventionsverhalten, der Risikowahrnehmung, der Maßnahmenbewertung und zum Vertrauen in verschiedenen Bildungsgruppen gewonnen. Die benannten Faktoren werden in der Forschung als maßgeblich für die Einhaltung von Eindämmungsmaßnahmen beschrieben (Health-Belief-Modell). Aufgrund begrenzter Datenverfügbarkeit konnte nur das erste Pandemiejahr bis zum Beginn der 2. Welle Ende Oktober 2020 untersucht werden.

Frau Dr. Ursula von Rüden (Bundeszentrale für gesundheitliche Aufklärung – BZgA) stellte die Ergebnisse der CoSid-Studie der BZgA dar. Die repräsentative Erhebung zu Wissen, Einstellung und Verhalten sowie Treibern und Barrieren der Inanspruchnahme der Corona-Schutzimpfung dient insbesondere der Kampagnensteuerung für Impfmaßnahmen. Zusammenfassend konnte aus den Daten abgeleitet werden, dass sozioökonomische Parameter auf der individuellen Ebene (z. B. Bildung, Einkommen, Migrationshintergrund) eine zentrale Rolle bei der Inanspruchnahme einer Corona-Schutzimpfung spielen. Ebenfalls als signifikant stellte sich die Rolle von Politikzufriedenheit und Vertrauen in behördliche Instanzen heraus [4, 5].

### Wohnungslose Menschen sind besonders vulnerabel in der Pandemie

Die Bedarfe von Menschen ohne Obdach und Wohnung waren das Thema der ersten Stunde des Kongresses, als dieser vor 27 Jahren ins Leben gerufen wurde. Bis heute wird die Debatte von den damaligen Initiator*innen Prof. Dr. Gerhard Trabert und Frau Dr. Jenny de la Torre begleitet. Neben neuen Daten und Praxisberichten gibt es jedes Jahr ein Netzwerktreffen. An diesem sind Initiativen beteiligt, die sich für die gesundheitliche Versorgung von Menschen engagieren, die ohne Wohnung und Obdach sowie ohne Krankenversicherungsschutz leben.

Die ohnehin schwierigen Zugangsbedingungen für Menschen ohne Obdach zu Hilfe- und Versorgungsstrukturen haben sich in der Zeit der Pandemie noch einmal deutlich verschlechtert. In den Zeiten der Lockdowns sind Versorgungsstrukturen vollständig zusammengebrochen. Aus niedrigschwelligen Angeboten sind Angebote mit Hürden geworden. Aufgrund von Abstandsregeln und Begrenzungen bei der zulässigen Personenzahl sind wichtige Schutzräume weggefallen, Einrichtungen und öffentliche Orte wurden geschlossen und die #stayathome-Initiative bekam einen zynischen Beigeschmack, erklärte Frau Dr. Maria Goetzens (Elisabeth-Straßenambulanz, Caritasverband Frankfurt e. V.). Während der Pandemie suchen in der Elisabeth-Straßenambulanz täglich Menschen mit Gewalterfahrungen Hilfe und Unterstützung. Hier zeige sich auch die hohe Prävalenz von psychischen und somatischen Erkrankungen, chronischen Erkrankungen, Suchterkrankungen sowie Erkrankungen der Atemwege.

In den letzten 2 Jahren sind Wohnungslose und Obdachlose verstärkt in den Blick der Öffentlichkeit gerückt, insbesondere in ihrer Rolle bei der Übertragung von SARS-CoV‑2, sagte Andrea Hniopek (Caritasverband für das Erzbistum Hamburg e. V.). Franziska Bertram (Universitätsklinikum Hamburg-Eppendorf) erklärte, dass mit den Daten der NASPHI-Studie zur psychischen und somatischen Gesundheit aufgezeigt werden konnte, dass die mittels PCR-Tests bestätigten Infektionen bei Menschen ohne festen Wohnsitz in Hamburg seltener registriert wurden als in der Allgemeinbevölkerung. Demgegenüber konnte bei ihnen eine höhere Seroprävalenz verzeichnet werden. Für Andrea Hniopek ist es generell und nicht nur in Krisenzeiten notwendig, Menschen ohne Obdach und Wohnung als hilfsbedürftige Menschen in die Mitte der öffentlichen Wahrnehmung zu nehmen. Alle Beiträge im Themenfeld konnten aufzeigen, dass eine sichere Wohnsituation den größtmöglichen Schutz bietet, um zur Ruhe zur kommen, gesund zu bleiben oder zu werden.

## Klimaschutz ist Gesundheitsschutz

In verschiedenen Veranstaltungen beschäftigten sich die Teilnehmer*innen des Kongresses mit den Auswirkungen der Klimakrise. Einerseits wird im Health Care Climate Footprint Report der Fußabdruck des Gesundheitssektors mit einem Anteil von 4,4 % an den globalen Emissionen beziffert [6]. Andererseits seien im Gesundheitssektor schon jetzt die Auswirkungen des Klimawandels direkt spür- und messbar, so Frau Dr. Francesca Racioppi vom Europäischen Zentrum für Umwelt und Gesundheit der Weltgesundheitsorganisation. Paul Zubeil, Vertreter der Abteilung für europäische und internationale Angelegenheiten im Bundesministerium für Gesundheit, führte aus, dass Global Health so weit oben auf der deutschen Agenda stehe wie nie.

Wie mit den konkreten Auswirkungen des Klimawandels in deutschen Städten und Gemeinden umgegangen wird, diskutierten die Teilnehmenden in den 2 Veranstaltungen der Arbeitsgruppe gesundheitsfördernde Gemeinde- und Stadtentwicklung (AGGSE). Hitzeangepasste Stadtentwicklung und Gesundheitsförderung hängen dabei eng zusammen. Frau Prof. Dr. Henny Annette Grewe (Hochschule Fulda) präsentierte Studiendaten, die zeigten, dass unter den immer häufiger auftretenden Hitzesommern in Deutschland insbesondere chronisch erkrankte Menschen in prekären Lebenslagen leiden. In einer demografisch alternden Gesellschaft stelle sich diese Erkenntnis als besonders gravierend heraus. Zugleich steige in stark von Hitze betroffenen Gebieten der Welt auch das Risiko für Früh- und Totgeburten [7].

Der dringende Handlungsbedarf auf diesem Gebiet wurde auch anhand von Daten des RKI zur Übersterblichkeit durch Hitze deutlich gemacht [8]. Praxiseinblicke erhielten die Teilnehmenden hinsichtlich der Umsetzung von Hitzeaktionsplänen der Städte Erfurt und Mannheim. Vertreter*innen beider Städte stimmten in ihren Vorträgen mit Dr. Hans-Guido Mücke (Umweltbundesamt) überein, dass es vor Ort den politischen Willen brauche, das Thema auf die Agenda zu setzen und entsprechende Ressourcen in der Verwaltung zur Verfügung zu stellen. Hans-Guido Mücke berichtete über aktuelle Studienergebnisse, die zeigen, dass für eine erfolgreiche Umsetzung von Maßnahmen der stärkste Einfluss von motivierten Einzelpersonen ausgehe, gefolgt von Förder- oder Drittmitteln sowie von gesetzlichen Grundlagen oder politischen Beschlüssen [9].

## Digitalisierung aktiv und lebensweltbezogen gestalten

Jutta Allmendinger regte in ihrer Keynote dazu an, durch Fort- und Weiterbildung die Digitalisierung aktiv(er) mitzugestalten. Ängste, z. B. vor Jobverlust, gelte es abzubauen. Eine gemeinsame Datennutzung könne zu einer besseren Gesundheit, etwa bei der Entwicklung von Präventionsmaßnahmen auf Bevölkerungsebene, beitragen.

In der Veranstaltung „Digital bedingte gesundheitliche Ungleichheit – ein neues Konfliktfeld?“ stellte Frau Dr. Petra Schmidt-Wiborg eine sozialethische Perspektive auf die Bedingungen der digitalen Transformation zur dauerhaften Verwirklichung des Gemeinwohls dar. Die Digitalisierung sei durch die Pandemie stark beschleunigt worden. Die sozialethische Perspektive würde dringend benötigt, da die digitale Transformation auch Machtverhältnisse und Verteilungen von ökonomischen, sozialen und kulturellen Ressourcen verändere. Wem gehören Gesundheitsdaten? Wie können Algorithmen rassismussensibel lernen? Petra Schmidt-Wiborg rief die zivilgesellschaftlichen Verbände auf, sich stärker einzubringen und eine Charta zur chancengerechten Nutzung der Digitalisierung aufzustellen.

Dr. Tomas Steffens (Diakonie Deutschland) schloss sich dieser Forderung an. Die „erst schmerzlich vermisste, dann massive Digitalisierung des Gesundheitswesens“ bringe auch Gefahren mit sich. Dabei träfe die Digitalisierung auf eine Gesellschaft der gesundheitlichen Ungleichheit, in der (digitale) Gesundheitskompetenz ungleich verteilt sei. Tomas Steffens regte zu einem Perspektivwechsel an: „Nicht der Einzelne braucht Fähigkeiten, um ein komplexes Gesundheitssystem zu durchdringen, sondern das System muss Strategien bereitstellen, um der Komplexität der Menschen gerecht zu werden“ [10]. Aufgabe sei es auch, die digitale Prävention und Gesundheitsförderung nicht individuenbezogen, sondern verhältnis- und lebensweltbezogen auszurichten.

## Weitere inhaltliche Schwerpunkte des Kongresses 2022

Neben den in der Keynote angesprochenen Kernherausforderungen wurden weitere zentrale Themen auf dem Kongress besprochen: darunter z. B. Gesundheit von Kindern und Jugendlichen in der Pandemie, Kinderarmut, Public Mental Health, der ÖGD sowie der Health-in-All-Policies(HiAP)-Ansatz.

### Gesundheit von Kindern und Jugendlichen in der Pandemie

Zahlreiche Veranstaltungen setzten sich mit der Situation von Kindern und Jugendlichen im Verlauf der COVID-19-Pandemie auseinander. Insbesondere die Eindämmungsmaßnahmen standen hierbei im Vordergrund. Dabei zeigte sich auch bei den Kindern und Jugendlichen in puncto Pandemiebewältigung ein sozialer Gradient.

Mechthild Paul (Leiterin des Nationalen Zentrums Frühe Hilfen an der BZgA) hob hervor, dass die Bedürfnisse von Kindern und Familien in der Pandemie zu wenig und zu spät in den Blick genommen wurden. Die vorhandenen Studienergebnisse wurden beispielsweise in dem Endbericht der Interministeriellen Arbeitsgruppe „Gesundheitliche Auswirkungen auf Kinder und Jugendliche durch Corona“ zusammengefasst [11].

Frau Prof. Dr. Julika Loss (RKI) erklärte, dass es in Deutschland keine umfassende Studie gibt, die die vielschichtigen Auswirkungen der COVID-19-Pandemie auf die Kindergesundheit über die Zeit abbildet. Gründe seien u. a. die fehlende Infrastruktur zur Ad-hoc-Datenerhebung sowie die zunächst fehlende gesundheitspolitische Priorisierung der Kindergesundheit. Zum Teil mussten Untersuchungssurveys und Schuleingangsuntersuchungen sogar ausgesetzt werden. Erst im Verlauf der Pandemie konnten Studien aufgesetzt werden, u. a. die Corona-Kita-Studie. Es gab allerdings immer wieder Studien oder Sondererhebungen, die v. a. über onlinebasierte Erhebungen spezifische Aspekte der Kindergesundheit in der Pandemie schlaglichtartig beleuchteten, z. B. COPSY zur psychischen Gesundheit oder MoMo (Motorik-Modul-Studie) zur körperlichen Aktivität von Kindern und Jugendlichen.

Frau Prof. Dr. Ute Thyen (Kinder- und Jugendmedizin an der Universität Lübeck) machte deutlich, dass Kinder nur selten schwere COVID-19-Verläufe aufweisen, allerdings die Datenlage zu Long-COVID noch unklar sei. Sie unterstrich, dass in Deutschland ca. 2400 Kinder unter 18 Jahren eine primäre Bezugsperson durch eine Corona-Infektion mit Todesfolge verloren hätten. Zudem machte sie darauf aufmerksam, dass Kleinkinder die Welt vorwiegend unter Pandemiebedingungen kennengelernt hätten. Dies könne entwicklungsneurologische Folgen haben, wie beispielsweise Unsicherheiten in der Beziehungsentwicklung, es beeinträchtige die Emotionsregulation oder führe zu verminderter frühkindlicher Bildung v. a. bei sozial benachteiligten Kleinkindern.

Eine deutliche Folge der Maßnahmen zur Pandemiebekämpfung ist der Rückgang der Bewegungsaktivität bei Kindern und Jugendlichen. Dieser ist zwar in allen sozioökonomischen Gruppen sichtbar, am deutlichsten allerdings in der niedrigen. 70 % der Kinder mit Übergewicht, aber nur 27 % der Kinder mit Normalgewicht berichteten eine Gewichtszunahme, 49 % eine Verschlechterung der Motorik [12]. Julika Loss sieht in der Pandemie aber auch eine Chance: „Das Thema Gesundheitsverhalten war während der Pandemie so präsent in den Medien wie noch nie zuvor.“ Die gesteigerte Aufmerksamkeit gelte es nun aktiv zu nutzen und soziallagenbezogene Angebote zur Bewegungsförderung bereitzustellen.

Dass auch die mentale Gesundheit massiv beeinträchtigt ist, zeigen die Daten der COPSY-Längsschnittstudie, die die Auswirkungen und Folgen der COVID-19-Pandemie auf die psychische Gesundheit von Kindern und Jugendlichen in Deutschland untersucht. Es zeigt sich eine deutliche Zunahme von mentalen Problemen, Ängstlichkeit und depressiven Symptomen. Diese verstärkten sich noch einmal vom ersten zum zweiten Lockdown [13].

Unterstützungsangebote für Familien sind in der Pandemie von zentraler Bedeutung. Ute Thyen warb dafür, zu etablierten und bekannten Strukturen zurückzukehren, in denen sich alle sicher fühlen. Beispielsweise sei das Vertrauen in Kinder- und Jugendärzt*innen auch in Pandemiezeiten durchgehend hoch gewesen. Zudem bräuchten Kinder bis zum Alter von 3 Jahren und ihre Eltern einfache und klare Botschaften.

Elisabeth Schmutz (Institut für Sozialpädagogische Forschung Mainz gGmbH) ergänzte, dass Kinder- und Jugendhilfe zum Großteil Beziehungsarbeit sei. Die Kontaktbeschränkungen während der Pandemie hätten die Kinder- und Jugendhilfe daher in ihrem Kern getroffen. Dem erhöhten Krisenpotential von Familien hätten nur eingeschränkte Interventionsmöglichkeiten entgegengestellt werden können, z. B. verstärkte digitale Angebote. Inhaltlich war es häufig darum gegangen, den beruflichen und familiären Alltag neu zu strukturieren und an die pandemischen Gegebenheiten anzupassen. Dabei hatte sich die soziale Infrastruktur der Kinder- und Jugendhilfe bzw. der Frühen Hilfen als zentrale Unterstützungsstruktur zur Krisenbewältigung erwiesen. Elisabeth Schmutz plädierte insgesamt für eine langfristige und verlässliche Existenzsicherung der sozialen Einrichtungen und Dienste.

Mechthild Paul unterstrich: Dort, wo es schon gute Strukturen gäbe, könne auch die Krise besser bewältigt werden. Mit den zusätzlichen finanziellen Mitteln aus dem Aktionsprogramm „Aufholen nach Corona für Kinder und Jugendliche“ der Bundesregierung konnten viele Projekte auf den Weg gebracht werden. Nun sei die Frage, ob diese Angebote auch dauerhaft aufrechterhalten werden können.

### Kinderarmut als fortwährende Herausforderung

Neben pandemiebedingten Veränderungen sind andere Themen bereits lange Zeit von hoher Präsenz für die Kindergesundheit. So verzeichnet Berlin seit 20 Jahren hohe Armutsquoten für Kinder und Familien. Regine Schefels und Sabine Hübgen von der Berliner Senatsverwaltung für Bildung, Jugend und Familie stellten im Rahmen des Kongresses die Berliner Strategie gegen Kinder- und Familienarmut vor. Dieses gesamtstädtische Projekt zielt darauf ab, die vielfältigen vorhandenen Einzelmaßnahmen zur Armutsprävention und -bekämpfung nach dem Vorbild der Präventionsketten zu systematisieren und besser miteinander zu verzahnen [14].

Kinder und Jugendliche standen auch bei der Abschlussveranstaltung im Fokus. Der in 2021 verabschiedete Koalitionsvertrag „Mehr Fortschritt wagen“ gibt viele Impulse für zentrale gesellschaftliche Veränderungsprozesse, die auch die Lebensumstände von Familien verbessern. Ricarda Lang (Parteivorsitzende von Bündnis 90/Die Grünen) machte als eine der Rednerin*innen der abschließenden Podiumsdiskussion deutlich, wie sehr der aktuellen Regierung die Bekämpfung der Kinderarmut am Herzen liege und verwies auf konkrete Maßnahmen wie den Beschluss zur Einführung einer Kindergrundsicherung. Annette Berg (Stiftung Sozialpädagogisches Institut Berlin – SPI) verwies auf ein im Dezember 2021 vom SPI und der Prognos AG veröffentlichtes Positionspapier „Perspektiven für die Kinder und Jugendpolitik im investierenden Sozialstaat“. In diesem fordern sie eine ebenen- und ressortübergreifende Koordination der Kinder- und Jugendpolitik, denn: „Um Armutsrisiken und -folgen bei Kindern und Jugendlichen zu reduzieren und gesellschaftlichen Zusammenhalt zu stärken, ist eine nationale Präventionsstrategie notwendig“ [15].

### Public Mental Health: neue Entwicklungen und Herausforderungen

„Public Mental Health [erfährt], gerade auch während der Pandemie, ein zunehmendes sowohl nationales als auch internationales Interesse“, führte Prof. Dr. Ulrich Reininghaus vom Zentralinstitut für Seelische Gesundheit in die Veranstaltung „‚Shifting the Curve‘: Neue Entwicklungen und Herausforderungen im Bereich der Public Mental Health“ ein. Diese wurde von der Deutschen Gesellschaft für Public Health ausgerichtet, die erneut Mitveranstalter des Kongresses war. Da die 12-Monats-Prävalenz von psychischen Erkrankungen aktuell bei 27,8 % liegt (Reininghaus), scheint das Interesse am Thema angemessen zu sein. Frau Dr. Julia Thom (RKI) unterstrich die Wichtigkeit der Forschung auf diesem Gebiet. Aktuell wird am RKI eine Mental Health Surveillance am Vorbild der Diabetes Surveillance aufgebaut, um systematisch entlang fest definierter Indikatoren und Handlungsfelder Daten zu liefen. Auch die bisher vernachlässigten Handlungsfelder Prävention und Gesundheitsförderung sollen berücksichtig werden.

Prof. Dr. Georg Schomerus (Universitätsklinikum Leipzig) ging auf die Stigmatisierung von Menschen mit psychischen und psychiatrischen Erkrankungen ein. Durch Kampagnen wie „time to change“ im Vereinigten Königreich habe sich die Einstellung gegenüber psychischen Erkrankungen in den letzten Jahren verbessert. Diese würden von der Gesellschaft inzwischen mehr als Teil ihres Alltags betrachtet. Gleichzeitig hätte sich jedoch das Konzept psychischer Erkrankungen „verbreitert“ mit der Folge, dass Gefühle wie etwa temporäre Trauer nicht mehr als gesunde Reaktion, sondern fälschlicherweise als Erkrankung wahrgenommen würden. Die Zunahme an Wissen führe dazu, dass bestimmte Krankheitsbilder, z. B. Depressionen, besser akzeptiert würden. Nachholbedarf gäbe es jedoch noch bei vielen weiteren Erkrankungen (z. B. Schizophrenie).

### Der ÖGD auf dem Weg zum modernen Public-Health-Dienst

Bei der dem Kongress vorangestellten Satellitentagung am 21.03.2022 war die mögliche Ausgestaltung eines modernen ÖGD diskutiert worden. Unter anderem soll diese mit Mitteln aus dem 2020 beschlossenen ÖGD-Pakt finanziert werden, welche für die personelle Aufstockung, Digitalisierung und Vernetzung der Gesundheitsämter in Deutschland vorgesehen sind. Die ausführliche Dokumentation zur Veranstaltung findet sich auf der Webseite des Kooperationsverbundes Gesundheitliche Chancengleichheit.^2^

Auch während der 3 folgenden Kongresstage fokussierten verschiedene Veranstaltungen den ÖGD und die Erwartungen an den Pakt für den ÖGD. Frau Dr. Nicole Rosenkötter (Akademie für Öffentliches Gesundheitswesen in Düsseldorf) berichtete von verschiedenen Diskussionsrunden mit Mitarbeiter*innen der Gesundheitsämter, die im Rahmen von Fachforen von der Akademie für Öffentliches Gesundheitswesen in Düsseldorf organisiert wurden. Die Vertreter*innen sehen in dem Pakt eine Chance, notwendige Veränderungen anzuschieben. Die Paktmittel können als Motor für die Etablierung von (digitalen) Innovationen und für die personelle Stärkung genutzt werden, auch um zukünftig agiler handeln zu können. Chancen liegen aber auch darin, Kooperationen (u. a. mit der Wissenschaft) zu stärken und den ÖGD insgesamt als attraktiven Arbeitgeber und kommunalen Akteur sichtbar zu machen. Viele Gesundheitsämter hoffen, sich durch die Paktmittel breiter ausrichten zu können. Hierzu gehört auch die Stärkung der Gesundheitsberichterstattung, der Gesundheitsförderung und Prävention. Mehrfach wurde in den Diskussionsrunden aber auch die Sorge thematisiert, dass notwendige Modernisierungsmaßnahmen nicht innerhalb der Paktlaufzeit geschafft werden können und wenig Zeit bleibt, um gut qualifiziertes Personal zu finden und langfristig an den ÖGD zu binden.

Innerhalb der Gesundheitsämter muss die Modernisierung weiter vorangetrieben werden, wobei ein ständiger Austausch über die Zielstellungen bundesweit und mit relevanten Stakeholdern sowie politischen Akteur*innen erfolgen sollte. Gesundheit müsse als Querschnittsaufgabe (Health in All Policies) verstanden werden, es sollten aber auch eine Diskussion über die „Leitplanken“ zur strategischen Ausrichtung des ÖGD und die Definition von Qualitätsmerkmalen erfolgen. Dabei stellt sich allerdings die Frage, von wem und wo diese definiert werden und ob ggf. konkretere Vorgaben in den Gesundheitsdienstgesetzen benötigt werden.

### Health in All Policies weiter etablieren

Gesundheit als feste Konstante in allen Politikbereichen mitzudenken, ist eine grundlegende Forderung des Kongresses. In diesem Jahr wurde der Stadtstaat Bremen als Beispiel gelingender Praxis vorgestellt. Bremen sei das erste deutsche Bundesland, welches den Health-in-All-Policies-Ansatz seit 2019 fest im Koalitionsvertrag verankert habe. Diese politische Grundsatzentscheidung spiegele sich in der „Zukunftskommission 2035“ des Bundeslandes wider, in der formuliert ist: „Quartiere sollen so gestaltet werden, dass sie ein gesundes Leben ermöglichen und fördern.“ Konkrete Umsetzungsschritte seien beispielsweise der Beitritt zum Gesunde-Städte-Netzwerk, zum Kooperationsverbund Gesundheitliche Chancengleichheit sowie der Aufbau eines eigenen Teams für die Themen Gesundheitszentren und Prävention. Dies mündete bereits in einer ressortübergreifenden Zusammenarbeit insbesondere bei den Themen Wohnen, Bildung und Soziales. Derzeit würden Gesundheits- und Hebammenzentren (z. B. LIGA – Gesundheitszentrum Gröpelingen) in Stadteilen mit einer Unterversorgung an Hebammen aufgebaut. Zudem erläuterte Sonja Wagener (Freie Hansestadt Bremen), wie als Reaktion auf einen hohen sozialen Gradienten beim Corona-Infektionsgeschehen in den Stadteilen Gesundheitsfachkräfte an Bremer und Bremerhavener Grundschulen in Quartieren mit besonderem Entwicklungsbedarf eingesetzt worden sind. Eine enge Koppelung der Fachkräfte an etablierte soziale Strukturen hatte dazu geführt, dass sie dort auch zu anderen Aspekten von Prävention und Gesundheitsförderung informieren konnten. Auch in puncto Impfungen setze Bremen auf zugehende, bedarfsgerechte Angebote und erziele damit hohe Impfquoten bei der Corona-Schutzimpfung. Doch wie bei allen Projekten sei auch hier zum Teil die durchgehende Finanzierung ein Problem.

Ein politischer Meilenstein war die Verabschiedung des Gesetzes zur Stärkung der Gesundheitsförderung und Prävention (Präventionsgesetz – PrävG) im Jahr 2015. Ziel des Gesetzes war es, Prävention und Gesundheitsförderung finanziell, strukturell und qualitativ zu stärken sowie die Koordination und Kooperation der Akteur*innen zu verbessern. Breite Zustimmung von Expert*innen erfuhr insbesondere die Ausrichtung des Gesetzes auf gesundheitsförderliche Lebenswelten (Verhältnisprävention mittels des Setting-Ansatzes) und auf die stärkere Zusammenarbeit und Bündelung aller Präventionsakteur*innen. An der Umsetzung gab es allerdings einige Kritik. Daher wurde bereits im Herbst 2021 ein Rahmenpapier für die Novellierung des Gesetzes formuliert, an dem der Kongress federführend beteiligt war [16]. 6 zentrale Thesen wurden im Papier formuliert:

- Aktivitäten in Lebenswelten im Rahmen der §§ 20a und 20b SGB V sollten kassenübergreifend und außerhalb einer Kassenkonkurrenz erfolgen.
- Die Nationale Präventionskonferenz müsse im Sinne des HiAP-Ansatzes neu ausgerichtet werden und alle notwendigen Stakeholder miteinbeziehen.
- Unabhängige Qualitätssicherung und Evaluation seien zu gewährleisten, aus den Ergebnissen müsse prozesshaft gelernt werden.
- Beteiligung des Öffentlichen Gesundheitsdienstes (ÖGD) an der Prioritätensetzung, Steuerung und Umsetzung;
- Beteiligung des geplanten Bundesinstituts für öffentliche Gesundheit;
- HiAP-Bezug, um kompensatorische Arbeit hin zu einer präventiven Arbeit in einem lernenden Prozess zu entwickeln.

Auch Johannes Wagner, MdB (Bündnis 90/Die Grünen), formulierte eindrücklich: „Prävention steht im Sondierungspapier an erster Stelle.“ Thomas Altgeld (Landesvereinigung für Gesundheit und Akademie für Sozialmedizin Niedersachsen) verwies auf die Frühen Hilfen als ein gutes Beispiel für die Zusammenarbeit verschiedener Akteur*innen im Rahmen der Gesundheitsförderung auf Bundes- und Landesebene.

Wie zivilgesellschaftlicher Einfluss für mehr gesundheitliche Chancengleichheit ausgeübt werden kann, war die zentrale Frage einer Veranstaltung, die sich mit der Rolle der Public-Health-Community beim sozialen Wandel beschäftigte.^3^ Frau Dr. Julia Gabler (Hochschule Zittau/Görlitz) verdeutlichte, dass zivilgesellschaftliche Aufgaben mehr umfassen als Dienstleistungen wie Fürsorge und Hilfeleistungen, sondern auch etwa Kritik oder Protest. Das Zukunftsforum Public Health und das Nachwuchsnetzwerk Öffentliche Gesundheit (NÖG) stellten sich als zivilgesellschaftliche Akteur*innen vor und reflektierten die eigene Rolle, Motivation und Vorgehensweise. „Public Health ist gerade dabei, politisch sichtbarer zu werden“, stellte Maike Voss (Klug – Deutsche Allianz Klimawandel und Gesundheit) fest. Denn die Pandemie hat nicht nur das Thema öffentliche Gesundheit und lange bekannte Schwachstellen, wie die starke Ungleichverteilung von Gesundheitschancen, stärker in das Licht der Öffentlichkeit gerückt, sondern auch neue Möglichkeiten, wie die breite digitale Vernetzung und stärkere akteursübergeifende Zusammenarbeit vorangetrieben. Diese gelte es nun gut zu nutzen, damit sie am „Verhandlungstisch des Wandels“ wirksam werden können.

Die zentralen Ergebnisse des Kongresses sind in Infobox 2 dargestellt.

#### Infobox 1 Kongress „Armut und Gesundheit“ vom 22.–24.03.2022

- Veranstalter: Gesundheit Berlin-Brandenburg e. V. und zahlreiche Partner*innen
- Webseite: www.armut-und-gesundheit.de
- Twitter: https://twitter.com/kongress_aug
- Public-Health-Podcast: https://www.armut-und-gesundheit.de/podcast
- Kongress Dokumentation: https://www.armut-und-gesundheit.de/ueber-den-kongress/dokumentation-2022

#### Infobox 2 Zentrale Ergebnisse des Kongresses

- Frieden ist eine unverzichtbare Grundlage für Gesundheit.
- Die Zivilgesellschaft ist heute wichtiger denn je.
- Die Pandemie ist für gesundheitliche und soziale Ungleichheit ein Risiko/Verstärker. Insbesondere ohnehin benachteiligte Gruppen (Wohnungslose, sozioökonomisch benachteiligte Personen) sowie Kinder und Jugendliche sind besonders betroffen.
- Für den Öffentlichen Gesundheitsdienst (ÖGD), die Gesundheitsförderung und Wahrnehmung der Bedeutung von Gesundheitsstrukturen werden neue Möglichkeiten aufgezeigt, größere Sichtbarkeit zu erlangen und eine Modernisierung vorzunehmen.
- Die Lage von Kindern, Jugendlichen und Familien während der Pandemie wurde erst (zu) spät in den Fokus gerückt.
- Die Klimakrise wurde durch den Krieg in der Ukraine und die Pandemie in den Hintergrund gerückt, sollte uns aber weiter beschäftigen, da sie weitere Krisen/Kriege auslösen kann und schon jetzt gesundheitliche Nachteile erzeugt.

## Footnotes

1 Die 5 Handlungsfelder der Ottawa Charta für Gesundheitsförderung (1986): gesundheitsförderliche Lebenswelten schaffen, eine gesundheitsfördernde Gesamtpolitik entwickeln, gesundheitsbezogene Gemeinschaftsaktionen unterstützen, persönliche Kompetenzen entwickeln, die Gesundheitsdienste neu orientieren [2].

2 Webseite des Kooperationsverbundes Gesundheitliche Chancengleichheit: www.gesundheitliche-chancengleichheit.de/satellit-armut-und-gesundheit/satellit-2022/, Zugegriffen am 26.07.2022.

3 Die Veranstaltung „Die Public Health-Community als zivilgesellschaftliche Akteur*innen und ihre Rolle für sozialen Wandel“ ist online über die Kongresswebseite www.armut-und-gesundheit.de/ueber-den-kongress/dokumentation-2022 abrufbar und kann – ebenso wie die Eröffnungsveranstaltung mit der Keynote von Frau Prof. Jutta Allmendinger – in voller Länge angesehen werden. Zugegriffen am 29.07.2022.
